# When Hyperglycemia Moves the Body: A Case of Diabetic Striatopathy

**DOI:** 10.7759/cureus.108379

**Published:** 2026-05-06

**Authors:** Mohamad Mansour, Hasan Obeidat, Christopher J Haas

**Affiliations:** 1 Department of Internal Medicine, MedStar Health Georgetown University (Baltimore) Program, Baltimore, USA

**Keywords:** diabetic striatopathy, hemiballismus, hemichorea, hyperglycemia, neuroimaging

## Abstract

Diabetic striatopathy (DS) is a rare but distinctive neurological manifestation of poorly controlled diabetes mellitus, characterized by the onset of involuntary movements, commonly hemichorea or hemiballismus, and distinctive basal ganglia abnormalities on neuroimaging in the setting of hyperglycemia. Pathophysiology is still not fully understood. Prognosis is usually favorable with glycemic control.

We present a case of a 71-year-old Asian man with uncontrolled type 2 diabetes who developed progressive right-sided hemichorea-hemiballismus over three weeks. Initial neuroimaging was misinterpreted, delaying diagnosis. Upon re-evaluation, hallmark findings were recognized, including unilateral putaminal hyperdensity on computed tomography (CT) and corresponding T1-weighted hyperintensity on magnetic resonance imaging (MRI). The patient demonstrated significant improvement following glycemic optimization and neuroleptic therapy.

This case highlights the importance of recognizing the characteristic clinico-radiologic features of DS to ensure a timely diagnosis and initiation of appropriate therapy, which typically leads to an excellent prognosis.

## Introduction

Diabetic striatopathy (DS) is an uncommon but increasingly recognized neurological manifestation of poorly controlled diabetes mellitus. Its estimated prevalence is approximately 1 in 100,000, although the condition is likely underdiagnosed. DS predominantly affects older adults, is reported more frequently among individuals of Asian descent, and has a slight female predominance. Clinically, it most often presents with acute or subacute unilateral choreiform or ballistic movements, typically contralateral to the affected striatum [1,2].

The pathophysiology of DS remains incompletely understood. Proposed mechanisms include hyperglycemia-related hyperviscosity and ischemic or metabolic injury to the striatum, as well as altered neurotransmitter balance involving reduced gamma-aminobutyric acid (GABA) availability and increased dopaminergic activity [1,3]. Characteristic neuroimaging findings include contralateral striatal hyperdensity on computed tomography (CT) and hyperintensity on T1-weighted magnetic resonance imaging (MRI). Although DS is often reversible with glycemic correction, additional anti-chorea therapy may be required for symptomatic control [1,4].

Timely recognition is clinically important because delayed or missed diagnosis may prolong disabling movements, lead to unnecessary diagnostic testing, and delay appropriate metabolic and symptomatic treatment. This case highlights the diagnostic challenge of DS when imaging abnormalities are subtle and initially interpreted as negative. The educational value of this report lies in demonstrating the importance of careful clinico-radiologic correlation and vigilant review of CT and MRI findings in a patient with poorly controlled diabetes and hemichorea-hemiballismus.

Portions of this work were presented as a poster presentation at the ACP Maryland conference, held at St. Agnes Hospital on May 1, 2025, and as an oral presentation to the Clinical Problem Solvers on June 10, 2025.

## Case presentation

A 71-year-old Asian man with a history of uncontrolled type 2 diabetes mellitus treated with metformin, hypertension, hyperlipidemia, prior ischemic stroke, and chronic kidney disease, presented with a 3-week history of progressive involuntary movements involving the right upper and lower extremities. Vital signs were unremarkable, showing a temperature of 37 °C, heart rate of 74, respiratory rate of 19, blood pressure of 130/62 mmHg, and oxygen saturation of 98% on room air.

Neurological examination revealed irregular, non-rhythmic, non-suppressible choreiform movements with intermittent larger-amplitude flinging ballistic movements, consistent with right-sided hemichorea-hemiballismus. The movements were present at rest and worsened with voluntary activity. Aside from the right-sided hemichorea-hemiballismus, no additional focal neurological deficits were noted. Physical examination showed bruising of the right forearm, thought to be secondary to repeated trauma from the flinging movements. Given the patient’s history of prior ischemic stroke and new-onset unilateral hyperkinetic movements, the initial differential diagnosis included acute or subacute stroke, focal seizure activity, metabolic or electrolyte derangement, medication-induced movement disorder, and structural basal ganglia lesions. Medication reconciliation did not reveal a clear medication trigger.

Laboratory testing revealed hyperglycemia with serum glucose of 247 mg/dL without evidence of ketosis, hemoglobin A1C of 11.1%, mild hyponatremia of 133 mEq/L, mild hyperkalemia of 5.7 mEq/L, normal other electrolytes, creatinine of 1.98 mg/dL, anemia with hemoglobin of 7.9 g/dL, normal platelets, normal liver enzymes, and negative C-reactive protein (CRP) (Table 1).

**Table 1:** Laboratory measurements of blood parameters RBS: Random blood sugar, HbA1c: Glycosylated hemoglobin, CO2: Carbon dioxide/bicarbonate, AST: Aspartate transaminase, ALT: Alanine transaminase, CRP: C-reactive protein

Parameter	Value	Normal range
RBS	247 mg/dL	<140 mg/dL
HbA1c	11.1%	<5.7%
Sodium	133 mEq/L	135–145 mEq/L
Potassium	5.7 mEq/L	3.5–5 mEq/L
Chloride	102 mEq/L	96-106 mEq/L
CO2	20 mEq/L	22-28 mEq/L
Magnesium	1.8 mg/dL	1.7-2.2 mg/dL
Phosphorus	3.5 mg/dL	2.5-4.5 mg/dL
Calcium	8.9 mg/dL	8.5-10.5 mg/dL
Creatinine	1.98 mg/dL	0.7 to 1.3 mg/dL
AST	22 U/L	10-40 U/L
ALT	17 U/L	7-56 U/L
Hemoglobin	7.9 g/dL	12.5-15 g/dL
Platelets	231 (x10^9/L)	150-450 (x10^9/L)
CRP	0.3 mg/L	<1.0 mg/L

**Figure 1:**
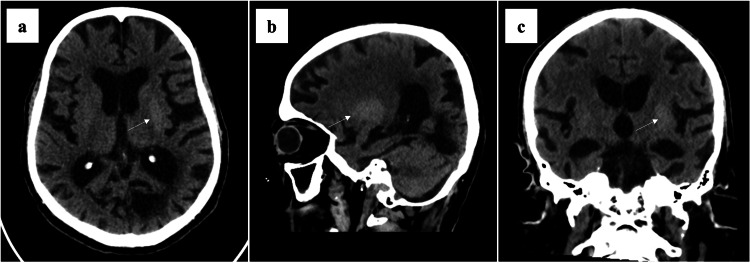
Axial (a), sagittal (b), and coronal (c) non-contrast brain CT images demonstrating subtle hyperdensity in the left putamen, contralateral to the patient’s right-sided hemichorea-hemiballismus, as highlighted by arrows CT: Computed tomography

**Figure 2:**
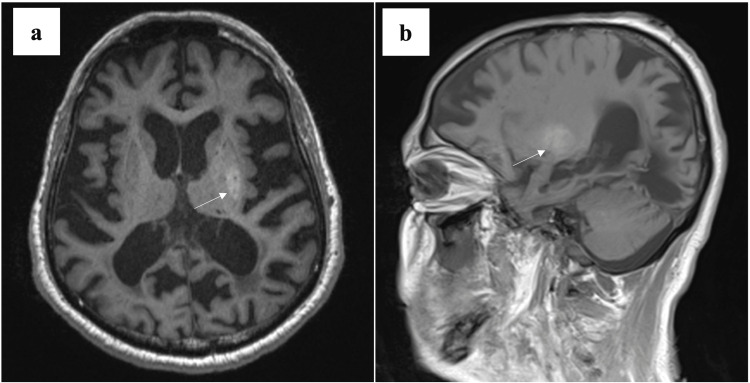
Axial (a) and sagittal (b) T1-weighted MRI demonstrating hyperintensity in the left lenticular nucleus, predominantly involving the putamen, as highlighted by arrows This finding corresponded to the left putaminal hyperdensity seen on CT and was compatible with diabetic striatopathy in the appropriate clinical context. MRI: Magnetic resonance imaging, CT: Computed tomography

Initial non-contrast CT and MRI of the brain were reported as negative for acute insults. They showed chronic ischemic changes with an area of encephalomalacia in the left occipital lobe, which is likely a result of an old insult. Electroencephalography (EEG) showed mild slowing of the normal posterior rhythm, suggestive of mild encephalopathy, most likely metabolic, with no epileptiform activity. The abnormal movement was not associated with any changes in the EEG. The patient was discharged with mild improvement of his symptoms after correction of metabolic derangements with an outpatient neurology follow-up appointment. The patient was seen in the clinic three weeks later and remained symptomatic. He was offered a benzodiazepine; however, he declined. Two days later, he presented to the hospital with worsening symptoms and developed new tongue protrusion. Repeat neuroimaging ruled out a new acute brain insult. Persistent symptoms prompted re-evaluation by a movement disorder specialist and careful re-review of the initial and repeat neuroimaging, at which point diabetic striatopathy was considered. Subtle but diagnostic findings were then identified, including hyperdensity of the left putamen on brain CT (Figure 1), and corresponding T1-weighted hyperintensity on brain MRI (Figure 2), findings consistent with DS. Review of the available MRI sequences beyond T1-weighted imaging showed no evidence of acute infarction or hemorrhage. The subtle striatal abnormalities were not initially recognized, likely because diabetic striatopathy was not included in the initial differential diagnosis, and the radiologic findings were overshadowed by chronic ischemic changes and prior left occipital encephalomalacia. After optimizing hyperglycemia with insulin and attempting as-needed haloperidol 5 mg and clonazepam 0.5 mg once a day for symptom control, the abnormal movements improved, and the patient was discharged to be followed up in the outpatient setting. However, unfortunately, the patient was then lost to follow-up.

## Discussion

This case illustrates the classic presentation and diagnostic challenges of DS. Compared with previously reported cases, our patient had typical features of diabetic striatopathy, including poorly controlled type 2 diabetes, Asian ancestry, unilateral hemichorea-hemiballismus, contralateral putaminal CT hyperdensity, and T1-weighted MRI hyperintensity. The distinguishing feature of this case was the diagnostic delay caused by subtle imaging findings initially interpreted as negative and by the absence of diabetic striatopathy from the initial differential diagnosis. This highlights the importance of considering diabetic striatopathy early in patients with poorly controlled diabetes and new-onset hemichorea-hemiballismus, even with only moderate hyperglycemia at presentation, and of carefully re-reviewing neuroimaging once acute infarction or hemorrhage has been excluded.

Although DS is commonly described by the triad of hyperglycemia, hemichorea-hemiballismus, and characteristic imaging findings, particularly a T1-weighted hyperintensity on brain MRI, it can be misdiagnosed due to its rarity, clinicians' unfamiliarity with it, the subtlety of the imaging findings, and sometimes discordance of clinico-radiological features [1,3,5]. Clinico-radiologic discordance is also well-described. Some patients might have chorea-like movements, with no imaging findings, while others might have typical imaging findings with no symptoms. Also, there are some reports of cases with unilateral neuroradiological lesions manifesting chorea in bilateral limbs, patients with bilateral striatal lesions showing unilateral manifestation of chorea, and others with unilateral chorea and ipsilateral striatal findings [2,3,4]. Due to this discordance, a classification of DS into symptomatic, clinically isolated, and radiologically isolated forms has been recently proposed [3].

This variability has contributed to uncertainty regarding the pathophysiology of DS. There are many suggested theories, of which three are the most popular: the metabolic, ischemia, and bleeding theories. Hyperglycemia and ischemia cause direct changes in the striatum, leading to altered GABA and dopaminergic neurotransmission. Each theory has its flaws and cannot explain all aspects of the disease [1-3,6-8]. These hypotheses are not mutually exclusive but rather depict interconnected mechanisms that collectively contribute to the development of this distinctive condition [8,9]. The imaging abnormalities have been explained by four main theories: petechial hemorrhage, mineral deposition like calcium, myelin destruction, and infarction with astrocytosis [1,3,4,9].

Management centers on two strategies. The cornerstone is prompt correction of hyperglycemia, which reverses the underlying metabolic disturbance and, in some cases, can solely lead to symptom resolution. Nevertheless, adjunctive pharmacologic therapy with anti-chorea drugs is needed in the majority of cases to provide symptomatic control of disabling movements. Antipsychotics, specifically haloperidol, and benzodiazepines are the mainstay of symptomatic therapy. Other medications, including dopamine-depleting agents, anticonvulsants, and serotonin reuptake inhibitors, were also used with variable success [1,4]. Prognosis is excellent in most cases. Clinical improvement typically precedes radiologic normalization, which may persist for weeks to months [2,4,5,10]. Recognition of this temporal dissociation helps avoid unnecessary repeat imaging or investigations.

## Conclusions

Diabetic striatopathy is a rare but reversible complication of poorly controlled diabetes that should be considered in patients with new-onset hemichorea-hemiballismus, even with only moderate hyperglycemia at presentation. Diagnosis requires careful clinico-radiologic correlation, as characteristic computed tomography and T1-weighted magnetic resonance imaging findings may be subtle and initially overlooked. Early recognition can prevent diagnostic delay and guide timely glycemic optimization and symptomatic treatment, which usually leads to an excellent prognosis. The lack of long-term follow-up in our case limits the assessment of sustained clinical and radiologic recovery.

## References

[1] Arecco A, Ottaviani S, Boschetti M, Renzetti P, Marinelli L (2024). Diabetic striatopathy: an updated overview of current knowledge and future perspectives. J Endocrinol Invest.

[2] Oh SH, Lee KY, Im JH, Lee MS (2002). Chorea associated with non-ketotic hyperglycemia and hyperintense basal ganglia lesions on T1-weighted MRI. A meta-analysis of 53 cases including four new cases. J Neurol Sci.

[3] Dubey S, Biswas P, Ghosh R, Chatterjee S, Kanti Ray B, Benito-León J (2022). Neuroimaging of diabetic striatopathy: more questions than answers. Eur Neurol.

[4] Chua CB, Sun CK, Hsu CW, Tai YC, Liang CY, Tsai IT (2020). "Diabetic striatopathy": clinical presentations, controversy, pathogenesis, treatments, and outcomes. Sci Rep.

[5] Xu Y, Shi Q, Yue Y, Yan C (2022). Clinical and imaging features of diabetic striatopathy: report of 6 cases and literature review. Neurol Sci.

[6] Chen Y, Wu C, Ren M, Wang Q, Wang Z, Zhang Y, Yu Y (2024). Clinical and neuroimaging characteristics of diabetic striatopathy: a case series report. Front Endocrinol (Lausanne).

[7] Zheng W, Chen L, Chen JH (2020). Hemichorea associated with non-ketotic hyperglycemia: a case report and literature review. Front Neurol.

[8] Yu L, Gu H, Hu W, Yang L (2025). Diabetic striate syndrome: an uncommon complication of diabetes requiring further conceptual expansion. Front Endocrinol (Lausanne).

[9] Chatterjee S, Ghosh R, Biswas P (2024). Diabetic striatopathy and other acute onset de novo movement disorders in hyperglycemia. Diabetes Metab Syndr.

[10] Wu X, Fu R, Yuan C (2026). Case series of 46 patients with nonketotic hyperglycemia-associated chorea: a retrospective follow-up study. J Clin Endocrinol Metab.

